# Parental Decision-Making for Infant Spinal Anesthesia – A Qualitative Study

**DOI:** 10.21203/rs.3.rs-7999271/v1

**Published:** 2025-11-20

**Authors:** Audrey Rosenblatt, Heather Ballard, Alexander B. Froyshteter, David I. Chu, Alina Lazar

**Affiliations:** Ann & Robert H. Lurie Children’s Hospital of Chicago; Ann & Robert H. Lurie Children’s Hospital of Chicago; Ann & Robert H. Lurie Children’s Hospital of Chicago; Ann & Robert H. Lurie Children’s Hospital of Chicago; Ann & Robert H. Lurie Children’s Hospital of Chicago

**Keywords:** Parent, Decision-making, Spinal, Anesthesia

## Abstract

**Background:**

Spinal anesthesia is a viable alternative to general anesthesia for infant surgical procedures below the umbilicus. However, choosing spinal anesthesia over general anesthesia is a complex medical decision for parents. Little is known about how parents perceive infant spinal anesthesia as an alternative to general anesthesia and how they make this decision for their children.

**Aims:**

The aim of this research study was to generate a holistic understanding of the parental decision-making process for spinal anesthesia in infants.

**Methods:**

A qualitative research study was conducted at a single, pediatric tertiary care institution from November 2023 – July 2024. Eligible participants were parents whose infants were scheduled for an outpatient urologic procedure and had been offered a choice between spinal and general anesthesia. Semi-structured interviews were conducted with parents while their child was in the operating room to create a contemporaneous picture of the parental decision-making process without the influence of procedural outcomes. Narrative data was analyzed using a constructivist grounded theory methodology. Health literacy was assessed with the BRIEF screening tool.

**Results:**

Twenty interviews were conducted with 32 participants – eight individual parent interviews and 12 parent dyads. Qualitative themes included the importance of: 1) the timeline of information attainment and processing, 2) cooperative decision-making with the subthemes of co-parenting and healthcare provider support, 3) the influence of maternal experiences with neuraxial anesthesia, 4) a desire for an anesthetic perceived to be less invasive, and 5) overall procedural risks with subthemes of neurotoxicity, respiratory complications, and spinal damage. Health literacy showed seven participants were marginal and 23 were adequate.

**Conclusions:**

Infant spinal anesthesia decisions are complex medical decisions for parents. Clear education, mental processing time, and guidance from anesthesia providers help parents make informed decisions.

## Introduction

Approximately six million pediatric patients undergo general anesthesia annually and 3.9 million have surgical interventions. (1, 2) Twenty-five percent of these patients are infants. (3) General anesthesia for children is statistically very safe, with the highest risk being demonstrated in infants and neonates. (4) However, pharmacologic research has led to concern about the long-term effects of general anesthesia on neurodevelopment. (3, 5) These concerns have sparked a resurgence of interest in spinal anesthesia a technique widely used in adult anesthesia as an alternative option without potential detrimental effects on the developing brain. (6–8) An increasing number of pediatric tertiary institutions currently offer spinal anesthesia as a safe and viable alternative to general anesthesia. (9)

Despite the rise in spinal anesthesia, parents’ perceptions and attitudes about choosing spinal or general anesthesia in infants are not well-characterized. Shared medical decision-making between parents and healthcare providers (HCPs) is informed by understanding the cognitive process and key concerns related to common decision pathways. (10–13) The option of spinal anesthesia has potential to add complexity to the decision-making process with anesthesia options being processed alongside surgical decisions. (14) By studying parental perceptions surrounding spinal and general anesthesia and their decision-making process, we can strive to help parents make more informed decisions about their child’s anesthetic care.

The aim of this research study is to generate a holistic understanding of the parental decision-making process for spinal anesthesia in infants as an alternative to general anesthesia, including the views of parents, the dynamics of their interactions with HCPs, and the shared decision-making process between parents and HCPs.

## Materials and Methods

This study adhered to the COnsolidated criteria for REporting Qualitative research (COREQ) to ensure methodology rigor (Supplemental File 2). (15)

### Study Design

This study used a qualitative design; semi-structured interviews were conducted using Charmaz constructivist grounded theory methods. (16) Qualitative semi-structured interviews were conducted on the day of surgery. Parents were interviewed while the child was in the operating room (OR) to create a contemporaneous picture of parental decision-making for spinal anesthesia not influenced by procedural outcomes.

#### Setting

Participants were recruited at a single, pediatric tertiary medical center in the Midwest of the United States from November 2023 – July 2024. All interviews were conducted in the private preoperative patient room.

#### Researcher reflexivity

Interviews were collected by AR; she is a Certified Registered Nurse Anesthetist and a PhD with formal training in qualitative interviewing and methods. HB, AL, and AF are physician anesthesiologists. DC is a pediatric urologist. AL, AF, and DC participate in these cases clinically. AR, HB, and AL participated in coding, code checking, and theme construction. All investigators work clinically at the site where this study was conducted. This allowed for insight into participant comments on workflows and procedures and additional context to parent comments during the interviews. AR has previous experience with anesthesia medical decision-making research. Charmaz’s constructivist ground theory emphasizes reflexivity rather than Glaserian neutrality, as such memo-writing and discussion within the research team during data collection and coding were employed to balance potential researcher bias and the influence of previous knowledge and assumptions.

#### Participants and procedures

This study included parents and legal medical decision-makers for infants who were eligible for spinal anesthesia at the study institution (i.e., less than 18 months old, undergoing outpatient urologic surgery lasting ≤ 90 minutes). The parent needed to be available during the infant’s surgical procedure, willing to be audio recorded, and able to converse in English or Spanish via an interpreter.

Participants were identified via the OR status board and through communication with the anesthesia team assigned to the case. All semi-structured interviews were conducted by AR. The interviewer had no clinical relationship or prior interaction with the participants. The interviewer approached the family in the preoperative space prior to the infant’s transfer to the OR. The interviewer identified herself as a CRNA present in a research capacity. She discussed study aims and parental eligibility for participation and provided written consent paperwork for the parent(s) to review. The interviewer returned after the infant was transferred to the OR and enrolled interested participants. The reimbursement for study participation was a $50 gift card. Participants signed written consent prior to the beginning of the interview. Interviews were conducted in the preoperative private room with the door closed. Interviews were audio recorded via Microsoft Teams and transcribed verbatim. Transcripts were de-identified at the point of transcription and the audio files were destroyed.

The semi-structured interview guide created for this study was adapted from the interview guide used in a study on parental decision-making for general anesthesia in young children and restructured to target decision-making for infant spinal anesthesia. (17) The interview guide focused on anesthesia choices and the decision-making process for spinal versus general anesthesia (Supplemental File 1). The interview guide was flexible and conversations were allowed to evolve naturally. The interview explored in-depth the topics parents initiated and lasted between 10 and 40 minutes.

### Data analysis

Data were analyzed using Charmaz constructivist grounded theory methodology. (16) Procedures outlined by Charmaz were used to structure this research and increase trustworthiness including: simultaneous collection of data and analysis, use of constant comparative techniques, analytic codes proceeding from the data, avoidance of a pre-existing hypothesis or theoretical structure, theory development advancing as each portion of data collection and analysis emerged, and the use of memo writing. (16, 18, 19)

Researchers applied the following steps to engage in the rigorous process outlined in the Charmaz constructivist method. Initial coding consisted of line-by-line coding to familiarize the researcher with the data and ground the research in the data (analytic codes proceeding from the data). (16) Data were analyzed after each interview and initial coding was applied (simultaneous collection of data and analysis). Previous transcripts were revisited after each new interview was initially coded (constant comparison) to examine data for codes which may have emerged with greater clarity as the data set expanded. (20) Focused coding was then applied to look for emergent themes and core categories which began to cluster together. Theoretical coding advanced the core concepts, ensured data saturation, and sharpened the analysis. Memo writing was used throughout this process and included notes on researcher thoughts and perceptions, modifications to previous codes, and ideas as core concepts began to emerge. Enrollment continued until thematic saturation was reached. Dedoose software was used to analyze narrative data. (21)

### Study Rigor

Trustworthiness in grounded theory is maintained through four criteria; credibility, originality, resonance, and usefulness. (16) Credibility was maintained through adherence to grounded theory methods during research conduct. All codes were reconciled in research pairs and code checking among the research team was performed through discussion of results as a wider team. Originality was achieved through the process of interviewer neutrality. The interviewer did not correct medical inaccuracies or provide additional medical information. She provided a reflective interview practice which explored what the participants understood without interjecting additional or new information which would have had the potential to alter the reported experiences. Resonance was achieved through interviewing processes which included reflexive practices including summarizing participant narratives within the interview structure and allowing for ample time for them to generate complete answers. Parent dyad interviews allowed participants to build on the responses of their co-parent which generated rich narrative data. Data saturation was achieved and participant sample size is robust. (22) Usefulness was ensured through researcher/clinician interaction as the data was collected. As the researchers are all clinicians within this clinical space, application of knowledge obtained was discussed. This research generated a range of actions such as increased outreach to local area pediatricians and ideas for next steps related to patient education.

## Results

The parents of 24 children were approached for participation. Twenty interviews were collected - eight individual interviews and 12 parent dyads. Thematic saturation was reached.

### Participant Demographics

The participant group was robust and represented a diverse cohort of age, gender, co-parenting pairs versus single participants, racial demographics, socioeconomic background, and health literacy (Table 1). Parents ranged in age from 23 to 46 years old. The focal patients were males aged six to 12 months presenting for urologic surgery. The 12 parent dyads were male and female co-parents. The eight individual interviews were two male and six female – five were parents present alone for the procedure, one was a participant whose co-parent was present but declined the interview, and three were present with grandparents who were not included in the interview. Five identified as Black/African American, one Hispanic/Latino, two Asian, and 24 participants identified as white/Caucasian. Zip codes were correlated to child opportunity index which ranged from Very High to Very Low. (23) Health literacy was assessed with the Basic Health Literacy Scale (BHLS) and scores ranged from 14 to 20 on a 20-point scale. (24) Seven participants had scores considered marginal (score 13–16) and 23 were adequate (score > 16; 2 missing data). Consistent with grounded theory methods, the goal is not statistical generalizability, but rather to capture the range of experiences from a diverse participant group which includes outlier information from exceptional or negative cases.

### Themes

Major themes included the importance of the: 1) timeline of information attainment and processing, 2) cooperative decision-making with the subthemes of co-parenting and healthcare provider support, 3) influence of maternal experiences with neuraxial anesthesia, 4) desire for an anesthetic perceived to be less invasive, and 5) overall procedural risks with subthemes of neurotoxicity, respiratory complications, and spinal damage (Table 2).

### Information Timeline

Participants felt receiving information ahead of time was both helpful and necessary for processing the information. Information about the option of spinal anesthesia prior to the day of the procedure allowed parents to research and understand spinal anesthesia. Parents received information about spinal anesthesia at a wide variety of points: ranging from the initial surgical consult appointment to the day of surgery with the majority receiving information via telephone a few days prior to the day of the procedure per institutional protocol.

This participant describes receiving information about spinal anesthesia during the surgical consult and the process of information gathering about spinal anesthesia.

“The doctor mentioned it when we did a consultation … So we’ve had a few months to think about it and figure out … what sources we were gonna use for getting the information we needed to make a decision.”

• Interview 11

These participants expressed gratitude that they were able to learn about spinal as an option ahead of time, even though one ultimately chose general anesthesia for her child.

I like that they were able to call me ahead of time and let me know instead of throwing it at me (the) day of, you know, cause I’m already a nervous wreck as it is and I wouldn’t want to be bombarded with more things on the spot.

• Interview 12

Participants described wanting information directly from the anesthesiologist in comparison to surgeons or other specialists for complete and robust information.

It’s (explanations from the surgeons about anesthesia) just not as good as an anesthesiologist can explain it, right? Like they’re just, they’re not anesthesiologists. That’s not their role, right?

• Interview 4

Participants used the terms; epidural, spinal tap, and spinal interchangeably emphasizing the need for time to provide guidance for a potentially poorly understood anesthetic choice.

(The anesthesiologist) just said basically that there were risks and benefits to both, but he seemed more reassuring that that the epidural. I keep calling it epidural, but we all know what I mean.

• Interview 13

### Cooperative Decision-Making

Parents described a cooperative interactive process between co-parents and HCPs, including support from the pediatrician and a connection with the anesthesiologist. They wanted information with the context of their child’s medical history from HCPs.

### Co-parenting

Trust was the most influential factor in cooperative decision-making between co-parents. Participants described a variety of experiences from primarily one parent independently making decisions to a highly collaborative process. Participants did not disclose any conflict around decision-making.

Participant 2: “We always verbally talk about (medical decisions). I tend to put a lot of my faith in her … (speaking to co-parent directly) because I hate to say it I put a lot on you. I work a lot … I’m really busy during the day so when I get out of work that’s when I look at it but”

### Participant 1: “He trusts my opinion.”

Participant 2: “I really trust her opinion. I don’t really question it because she’s a stay-at-home mom and these kids are her life … (speaking to co-parent directly) you are on top of it.”

• Interview 1

Participant 1: “We were there (together) for every appointment. … We were both aligned on this decision from an anesthesia standpoint.

Participant 2: “We were in the appointments together that helped. We kind of made the decision together pretty early on that we thought that this was the best path with the spinal tap.”

• Interview 9

### Healthcare Provider Support

Cooperative decision-making with HCPs was influenced by the timeline. Parents reached out within the healthcare system for additional information, primarily to their child’s pediatrician for medical recommendations.

I (had) actually not heard of (spinal anesthesia) either because even our pediatrician when I talked to him about it on Monday … he was like, oh I’ve never heard of that.

• Interview 4

The doctor (anesthesiologist) being very calm about it and very reassuring about it and even when I spoke to my son’s pediatrician, … it didn’t seem like there was a list of complications or anything dangerous about it.

• Interview 13

Written information was seen as helpful when it came from hospital resources, but parents wanted to discuss spinal anesthesia with a HCP.

I think maybe like if more people start saying yes to the spinal, maybe sending that like digitally beforehand, like just so you can read about it before you get here will be helpful. But I feel like the conversation was so necessary

• Interview 4

Internet research was regarded with skepticism or fear as the information lacked context to their individual situation and resulted in anxiety rather than information.

I’m not touching it (Reddit internet forums) because there’s no context, like you don’t know the situation (of) everything.

• Interview 17

I didn’t go … and Google, ‘what happens to … a baby’s brain on anesthesia? Or can a baby become paralyzed from a spinal?’ I didn’t look up anything because I didn’t want to scare myself.

• Interview 13

### Maternal Experiences with Neuraxial Anesthesia

Maternal experience with neuraxial anesthesia for childbirth was important. They leveraged their decision-making process from their childbirth experiences in this decision-making process.

Participant 1: “I think based on our research from pregnancies and going through labor and based on our research of having an epidural done and risks and side effects of that I guess we kind of associated the spinal block”

### Participant 2: “We remembered the risks of the epidurals”

Participant 1: “With … the way it was explained to us it seemed like it was very similar.”

• Interview 7

### Less Invasive Anesthetic

Spinal anesthesia was described as “less” intervention with a preference for “natural” breathing and sleep. There was hesitation around the invasive nature of the spinal, but this was seen as less invasive than the interventions associated with general anesthesia. Respiratory interventions and the medically induced unconsciousness associated with general anesthesia were both seen as more invasive.

Participant 1: “My impression is … it’s different like they put like a tube in and it’s just more”

### Participant 2: “invasive”

Participant 1: “Invasive, like a stronger medication. Where this other option (spinal anesthesia) … he would be able to take his pacifier, they would give him something to kind of chill him out, but he would naturally fall asleep vs kind of being forced to with the general anesthesia.”

• Interview 19

### Procedural Risks

Procedural risks included the balance of the risks between the two options with neurotoxicity and respiratory complications being most impactful. Participants described being uncomfortable but not being able to clearly elucidate the reasons for lack of comfort with the anesthesia type they excluded.

So they (the anesthesiologist) called us last weekend … here’s your other choice. Like, you have seconds to make the decision … and they told us about it. I really didn’t do research on it because I’m kind of gung-ho like, OK, we both (co-parents) are kind of gung-ho that we do not want to mess with his spine. He’s too little for that.

• Interview 12

This parent described a preference for spinal anesthesia but struggled to explain why it felt like a better choice. This tension was described by multiple participants.

Mostly from the research that I’ve done - the risk for a full-term baby, that has no health problems, the risk is relatively the same between general anesthesia and spinal. So, while it’s not really that different … it’s just like one of those things you can’t really like. I mean, I guess because he doesn’t have to have the breathing tube and the drugs for it.

• Interview 1

### Neurotoxicity

The potential for neurotoxicity was discussed by HCPs during informed consent, and it was critical to decision-making. This participant describes a worst risk versus worst risk as a deciding factor.

I look too at like worst case scenario would I rather have my baby’s brain be damaged or their body be damaged. And I was like, we’re gonna go with body, be damaged not the brain.

• Interview 13

This participant describes looking back at experiences with her older child when making anesthesia decisions and concerns about negative repercussions of previous decisions.

Our oldest son is 10 years old and he had an operation here at (hospital name) as well when he was born. They used general anesthesia … To this day, he still has some development issues when it comes to cognitive learning abilities …. We’ve always kind of wondered if … if that impacted his brain development in anyway. There’s obviously no way for us to know … we always second guess for ourselves. But with the spinal being available, that gave us another alternative.

• Interview 11

### Respiratory Complications

Airway instrumentation was seen as invasive and a deterrent to choosing general anesthesia.

We were like, okay, that sounds like the best option if we can avoid being more invasive and putting something down his throat and putting him under why not?

• Interview 5

This brought her experience with surgical complications to the decision-making process. This quote also highlights the requirement for grounded theory data saturation of unique, exceptional, or rare participant recruitment.

I work as a nurse in ICU but not with kids, so … I’m familiar with the situations … I’ve received couple of patients that is supposed to be just outpatient surgery and then we had to wait for a couple of days because they couldn’t extubate. So I’m just scared of this kind of complications.

• Interview 14

### Spinal Damage

Parents expressed significant concern about the risk to the spine and potential for nerve damage. This was particularly important as the small size and young age of the child was emphasized by the parents as an increased risk for damage. This quote also emphasized the crossover with maternal experiences related to labor and delivery neuraxial anesthesia.

I had an epidural and I don’t know if that’s one reason why I’m having some issues getting up every morning … I feel cramp(s), like my legs bother me. We don’t wanna mess with his spine since he’s so little. You don’t know what the effects are gonna be later on.

• Interview 12

### Conceptual Framework

**Figure 1:**
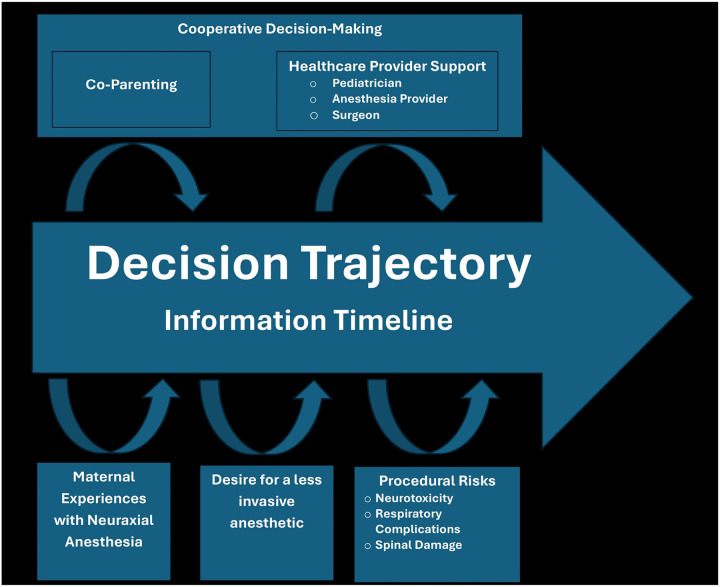
Theoretical Model Conceptual model of parental decision-making for infant spinal anesthesia. This figure represents the decision trajectory and the interaction of the major themes and subthemes in decision-making.

The conceptual framework (Fig. 1) constructed during theoretical coding provides a graphic view of the decision trajectory with the influence of the major themes and subthemes. The information timeline, or the point at which parents were aware of the option for spinal anesthesia, influenced the decision trajectory. Earlier notification allowed for a less compressed and more robust engagement with the information through support within a co-parenting framework and through discussion with healthcare providers including surgeons, anesthesia providers, and pediatricians. Given a longer timeframe for consideration, participants reached out to the surgeon for follow up information and the pediatricians for information and recommendations. Key informational factors included previous experience with neuraxial anesthesia, specifically the maternal experience with neuraxial anesthesia for labor and delivery. There was a desire for a less invasive anesthetic overall – this underlay the decision for either general anesthesia or spinal anesthesia with the final decision often being described as the less invasive choice on either side. The procedural risks described as most impactful to the decision were the risk of neurotoxicity and respiratory complications when choosing spinal anesthesia, and the risk of damage to the spine when choosing general anesthesia. There was overlap between the procedural risks and personal experience of neuraxial anesthesia; particularly when it was negative the risk of spinal anesthesia was considered to be higher.

## Discussion and Practice Implications

This research revealed five major themes within parental decision-making for infant spinal anesthesia; the importance of the timeline of information attainment and processing, the role of cooperative decision-making with co-parents and HCPs, the influence of maternal experiences with neuraxial anesthesia, a desire for an anesthetic perceived to be less invasive, and overall procedural risks.

The timeline of information sharing impacted the parents’ ability for shared decision-making (SDM) with HCPs. The findings of this study demonstrate some of the challenges of meaningful incorporation of parents into the anesthesia decision-making process. Parents need time to process information, and understanding the complexities of spinal anesthesia was time consuming, particularly when health literacy was low. (25) If SDM is a goal for anesthesia decision-making, there may not be enough time in the preoperative interview conducted on the day of surgery to accomplish this. (26) There is not a universal standard approach to the incorporation of parents or patients into anesthesia decision-making across practice settings and practice ranges widely from informational to shared decision-making. (14, 27) Parents prefer SDM for elective, outpatient procedures (the demographic for this research cohort) and anesthesia decision-making has potential to be included in this umbrella. (28)

Parents engaged in a cooperative process of decision-making with support from HCPs. The timing of the introduction of spinal anesthesia, when it occurs after the initial surgical visit, disrupts their expectation of general anesthesia as the primary anesthetic. Participants described reaching out to their child’s pediatrician to discuss spinal anesthesia options during surgical planning. Pediatricians provide decision-making support on a wide range of medical choices. (29) Participants described mixed reactions from pediatricians and for some a lack of awareness of spinal anesthesia as an option or knowledge of the risks or benefits of the procedure. Given the timeline of interaction with an anesthesia practitioner, education and outreach to pediatricians regarding infant spinal anesthesia will help support families with accurate information and resources. Outreach to primary care providers for education and as an opportunity for questions may foster the uptake of spinal anesthesia.

Health literacy was impactful in understanding the complexities within neuraxial anesthesia. (25) Spinal, epidural, and spinal tap were used interchangeably, representing a lack of understanding of the granular differences within neuraxial anesthesia. One participant described the anesthesiologist drawing the spinal cord and epidural space on the sheet of the crib mattress before they were able to understand the anatomy. Another participant initially told the researcher that they had chosen spinal when upon further conversation it became clear they had consented for general anesthesia with a caudal. These misunderstandings highlight the risk that those with lower health literacy may struggle to understand their choices. (25) Health literacy impacts trust in physicians and a combination of a highly complex medical decision combined with a lack of established relationship in a compressed timeline creates a unique decision-making circumstance. (30) Opportunities for the development of educational tools that address these misunderstandings and the underlying potential lack of anatomic understanding of the spinal cord are future possibilities to support parents with low health literacy. (31)

Parents discussed their prior decision-making pathway for labor neuraxial anesthesia as impactful for the spinal anesthesia context. Infant spinal anesthesia decision-making follows within months of mothers making choices for labor analgesia. Among women who decline neuraxial analgesia for labor management, there have been reported inaccurate understandings of the risk of the procedure. (32) Parents understandings or misunderstandings of labor analgesia are brought forward into their decision for infant spinal anesthesia. Additionally, positive experiences with labor analgesia (e.g. comfort, safety) and negative experiences (e.g. back pain, sensory disorientation) were considered in the decision-making process.

These findings have implications for how anesthesia providers, surgeons, and pediatricians present information regarding spinal anesthesia and how they support parents in the decision-making process. For medical centers interested in establishing infant spinal programs or those who are seeking to change the informational structure within a current program, these findings can information the process.

### Strengths and Limitations

Ground theory methodology is best applied when little is known about a topic, as it allows for freedom to explore a complex phenomenon and generation new knowledge rather than testing predetermined hypotheses. (16) There is little known about parental attitudes towards infant spinal anesthesia and as such this an ideal methodology to explore this topic. A qualitative grounded theory approach, through flexible methods and an inductive process, builds foundational understanding of the parents’ experiences and intellectual approach to the decision trajectory for infant spinal anesthesia. Qualitative methods, such as in-depth interviews, allow for the emergence of data which researchers might not consider asking about directly in a survey or via a previously validated tool. The grounded theory methodology utilized in this study is particularly beneficial as it keeps the analysis close to the narrative data so new knowledge can be formed which can inform both clinical practice and future research.

Qualitative research has inherent limitations including lack of generalizability of the data through findings which may be restricted to a specific context or participant group. However, the goal of qualitative research is resonance, not generalizability. (33) These limitations were mitigated through rigorous adherence to the grounded theory methodologic framework and continuation of data collection until thematic saturation was reached. Risk of bias was mitigated through adherence to methodologic standards to enhance trustworthiness in the qualitative methods including the use of memo writing and the use of independent coding, code reconciliation, and multiple coders.^18^ This study was limited to outpatient urologic procedures. Parents described varying levels of medical complexity, however patient data was not verified via chart review. All participants were interviewed in English. The study protocol did not collect native language or country of origin, although the interviewer identified at least five families where English appeared to be a non-native language.

## Conclusions

Parents need HCP support to understand their choices for anesthesia and the risks and benefits of general versus spinal anesthesia. They need time and space to process information and make decisions. The timeline for how spinal anesthesia information is shared with families will influence their decisionmaking trajectory. Parental experiences related to neuraxial anesthesia for labor and delivery are recent and relevant experiences when parents are making choices for their infants related to spinal anesthesia. Exploring the attitudes towards those experiences may help clinicians understand the context that parents bring to the decision. Parents make decisions within a supportive framework through cooperative decision-making with each other and with support from HCPs. Setting up these expectations early within the surgical planning, outreach to pediatricians and surgeons to ensure those in primary care and presurgical planning are well informed, and openness to dialogue and support are all critical elements for supporting parental decisions for spinal anesthesia.

### Future Directions

Future research should explore best practices for education regarding neuraxial techniques. There is complexity with this anesthesia technique which requires a high level of health literacy. This research highlighted the importance of additional decision-making time and expanded explanation of the spinal technique. Evidence-based strategies for parent education and assessment of understanding can aid families in decision-making and improve efficiency in practitioner/parent interactions.

## Supplementary Material

Supplementary Files

This is a list of supplementary files associated with this preprint. Click to download.

- SupplementalFileInterviewGuide.docx
- SupplementalFileCOREQ.docx

## Figures and Tables

**Table 1:** Participant Demographics (N = 32 parents, N = 20 interviews)

Category	Participants N (%)
Sex	Female 18 (56%)
	Male 14 (44%)
Focal child sex	Female 0 (0%)
	Male 20 (100%)
Age	Max 47 years
	Minimum 23 years
	Median 35
	Average 35.1 years
	Missing data 2 participants (1 interview)
Race/ethnicity	Black/African American (not Hispanic) 5
	Hispanic/Latino 1
	Asian 2
	Caucasian/white 24
Child Opportunity Index	Very high 9 (5 interviews)
	High 8 (5 interviews)
	Moderate 2 (1 interview)
	Low 9 (7 interviews)
	Very low 2 (1 interview)
	Missing data 2 participants (1 interview)

**Table 2:** Major themes and subthemes

Major Theme	Subthemes	Quotes
Information timeline		Participant 1: *“Yeah because like when we first heard it, it was kind of like, wait, what? You know, like you’re gonna do what?”* Participant 2: *“Yeah, which is great that the doctor mentioned it when we did a consultation* … *So we’ve had a few months to think about it and figure out who we were, what sources we were gonna use for getting the information we needed to make a decision.”* – Interview 11
Cooperative decision-making	Co-parenting	Participant 1: *“We bounced it back and forth”* Participant 2: *“We are both analytical people”* Participant 1: *“We both said which one we kind of preferred and it ended up being the same one”* – Interview 7 Note – this couple engaged in cooperative overlapping throughout the interview with sentence-by-sentence variation between speakers – each one finishing the other’s sentences.
	Healthcare provider support	*“She (the anesthesiologist) drew a picture for us and she explained it doesn’t go directly into the spine it’s kind of in the sac around it so her explaining gave us a better understanding of exactly where it would be placed in the baby and where it would be.”* – Interview 7
Maternal experiences with neuraxial anesthesia		*“You know, each time they gave her (mom) the epidural, it was like this kind of tense thing.” “The anxiety was the highest for me at that time … I knew what to expect (for the epidural), pain and all or a pinch or whatever … but I’m not six months (old).”* – Interview 18
Less invasive anesthetic		*“It just … sounds so invasive like, you know, into the spinal, you like specific sections of the spine”* – Interview 18
Procedural risks	Neurotoxicity	*“They have not seen in humans, that there’s brain issues from too much anesthesia at a young age, but they have seen a little bit in animals, and so I was just like – if I can steer clear of it I’d rather.”* – Interview 4
	Respiratory complications	*“I don’t know why it just it sounds scary to me. Like I just think not breathing on your own. It’s just like, so not ideal. It just sounds like there’s another complication that couldn’t potentially happen.”* – Interview 4
	Spinal damage	*“(I asked questions about) side effects or back pain for his future. Because I’ve got an epidural and it’s been six months and I still have back pain.”* – Interview 10

## Data Availability

The qualitative data supporting the findings of this study are not publicly available due to concerns regarding participant confidentiality and privacy.
